# Supplementary material for: Critically Ill Patients with Newly Diagnosed Anti-Neutrophil Cytoplasmic Antibody-Associated Vasculitis: Case Series and Literature Review
Source: J Clin Med. 2024 Sep 25;13(19):5688. doi: 10.3390/jcm13195688 (PMC11477170; doi:10.3390/jcm13195688)
Supplement: Supplementary file 1 [file jcm-13-05688-s001.zip › Rukavina et al.2024_SupplementaryS1_rev_24_09.pdf]

Table S1: Patient no. 1 summary

General data	• 58-year-old female Caucasian
Prior medical history	• None
Recent medical history	• Chronic cough for 3 months prior to admission<br>• Hemoptysis, dyspnea and chest pain one week prior to admission
Initial relevant clinical findings	• Dyspneic and fatigued<br>• Skin pallor, no rash<br>• Bilateral lung crepitations with diminished respiratory sounds
Initial relevant lab. results	• PCT 3 ug/L, CRP 124 mg/L<br>• RBC: $2.52x10^9/L$<br>• Urea 54 mmol/L, creatinine 1406 umol/L<br>• Urinalysis: E+++, proteinuria++
ANCA positivity	• MPO-ANCA
Initial relevant imaging results	• Chest CT scan: Right lung almost completely consolidated with peribronchal ground glass opacities; massive areas of consolidations of the left lung with diffuse ground glass opacities<br>• Bronchoscopy: Positive for DAH
Kidney biopsy	• Not performed
Ventilatory support	• MV → VV-ECMO → MV
Renal replacement therapy	• CRRT
AAV-remission induction therapy	• Glucocorticoids (3 days MP 1000 mg iv., 7days 2 mg/kg IV , afterwards 0.5-1 mg/kg IV)<br>• CYC (15 mg/kg IV; TD: 3000 mg)<br>• RTX (1000 mg iv. per dose; TD: 2000 mg)<br>• Intravenous immunoglobulins (2 mg/kg IV; TD: 160 g)<br>• Plasma exchange
Infectious complications	• Sepsis
Outcome	• Deceased; In-ICU death due to sepsis-induced MOF.
Follow-up	• N/A
Follow-up BVAS score	• N/A

Abbreviations: CRP: C-reactive protein; PCT: procalcitonin; GGO: ground glass opacities; NRM: non-rebreather mask; NC: nasal cannula; HFNC: high flow nasal cannula; MV: mechanical ventilation; CRRT: continuous renal replacement therapy; SOFA: Sequential Orfan Failure Assessment; BVAS: Birmingham Vasculitis Severity; MP: methylprednisolone; CYC: cyclophosphamide; RTX: rituximab; AAV: ANCA-associated vasculitis; TD: total dose; Urinalysis (E, LE, Prot) was performed by dipstick method and the grading system is as follows: "negative", trace ("+/-"), positive/detectable ("+"), moderate ("++"), high grade ("+++").
