# Supplementary material for: Critically Ill Patients with Newly Diagnosed Anti-Neutrophil Cytoplasmic Antibody-Associated Vasculitis: Case Series and Literature Review
Source: J Clin Med. 2024 Sep 25;13(19):5688. doi: 10.3390/jcm13195688 (PMC11477170; doi:10.3390/jcm13195688)
Supplement: Supplementary file 1 [file jcm-13-05688-s001.zip › Rukavina et al.2024_SupplementaryS2_rev1309.pdf]

Table S2: Patient no. 2 summary

General data	• 64-year-old male Caucasian
Prior medical history	• CKD stage 3B diagnosed approximately 6 months prior to admission<br>• Arterial hypertension<br>• Aneurysm of the infrarenal segment of abdominal aorta<br>• Intermittent macrohematuria for the past several years
Recent medical history	• Progressive dyspnea with intermittent hemoptysis and progressive exertional intolerance for 2-3 months prior to admission; the symptoms began after a COVID-19 infection<br>• Fatigue and low-grade fever (< 38 °C)
Initial relevant clinical findings	• Dyspneic (SpO2 78% with 15 L/min O2 via NRM)<br>• Lungs: Right crackles, left rales
Initial relevant lab. results	• CRP 237 mg/L<br>• Hgb 66 g/L<br>• Urea 31.2 mmol/L, creatinine 660 umol/L<br>• Urinalysis: E+++, proteinuria+
ANCA positivity	• MPO-ANCA
Initial relevant imaging results	• Chest CT: Diffuse bilateral interstitial infiltrates with areas of ground glass opacities
Kidney biopsy	• Moderate chronic tubulointerstitial and glomeruli changes with joined crescentic glomerulonephritis and signs of acute tubular injury. These changes are indicative of AAV
Ventilatory support	• NC/NRM → HFNC (FiO2 78%) → MV → NC → None
Renal replacement therapy	• CRRT during the first week of ICU stay
SOFA score	• 14
Initial BVAS score	• 18
AAV-remission induction therapy	• Glucocorticoids (3 days MP 500 mg IV, 7 days 1 mg/kg IV then tapered to oral doses)<br>• CYC (15 mg/kg IV per dose; TD: 6000 mg iv.)
Infectious complications	• None.
Outcome	• Remission
Follow-up	• After induction with CYC no clinical signs of active AAV<br>• CKD 3B<br>• He is due for RTX 2x1000 mg IV then maintenance with 500 mg every 6 mths
Follow-up BVAS score	• 5

Abbreviations: CRP: C-reactive protein; PCT: procalcitonin; GGO: ground glass opacities; NRM: non-rebreather mask; NC: nasal cannula; HFNC: high flow nasal cannula; MV: mechanical ventilation; CVVHD: continuous veno-venous hemodialysis; SOFA: Sequential Orfan Failure Assessment; BVAS: Birmingham Vasculitis Severity; MP: methylprednisolone; CYC: cyclophosphamide; RTX: rituximab; AAV: ANCA-associated vasculitis; TD: total dose; Urinalysis (E, LE, Prot) was performed by dipstick method and the grading system is as follows: "negative", trace ("+/-"), positive/detectable ("+"), moderate ("++"), high grade ("+++").
