# Supplementary material for: Critically Ill Patients with Newly Diagnosed Anti-Neutrophil Cytoplasmic Antibody-Associated Vasculitis: Case Series and Literature Review
Source: J Clin Med. 2024 Sep 25;13(19):5688. doi: 10.3390/jcm13195688 (PMC11477170; doi:10.3390/jcm13195688)
Supplement: Supplementary file 1 [file jcm-13-05688-s001.zip › Rukavina et al.2024_SupplementaryS3_rev_24_09.pdf]

Table S3: Patient no. 3 summary

General data	• 46-year-old female Caucasian
Prior medical history	• Recurrent epistaxis and middle ear infections within the past several years
Recent medical history	• Progressive exertional intolerance a month before admission<br>• Hemoptysis for two weeks before admission
Initial relevant clinical findings	• Day 1: Tachypnea 20/min, SpO2 98%<br>• Day 16: Dyspnea (SpO2 68% while breathing ambient air); fever > 38°C; bilateral lung rales
Initial relevant lab. results	• CRP increase from 7.1 to 269.3 mg/L<br>• PCT 0,16 ug/L<br>• Hemoglobin 116 g/L<br>• Urea 4,7 mmol/L, creatinine 71 umol/L<br>• Urinalysis: massive erythrocyturia, borderline proteinuria (semiquantitative)
ANCA positivity	• PR3-ANCA
Initial relevant imaging results	• Rhinoscopy: Vulnerable nasal mucosa with chronic atrophic changes<br>• Paranasal sinus CT: Maxilarry and sphenoid sinusitis<br>• Chest CT: Progressive bilateral lung GGO and consolidates
Kidney biopsy	• Not performed
Ventilatory support	• NRM (8 L/min) →NC →None
Renal replacement therapy	• None
SOFA score	• 3
Initial BVAS score	• 20
AAV-remission induction therapy	• Glucocorticoids (3 days MP 1000 mg IV, 7 days 2 mg/kg IV, then tapered to oral dosing)<br>• RTX (375 mg/m²/IV per weekly dose, TD: 2600 mg)
Infectious complications	• None
Outcome	• Remission
Follow-up	• One month after concluding induction therapy she is w/o clinical signs of AAV, with normal inflammatory markers and renal function<br>• Erythrocyturia <10 RBC/hpf persists<br>• Oral methotrexate was introduced and RTX will be combined as maintenance therapy
Follow-up BVAS score	• 0

Abbreviations: CRP: C-reactive protein; PCT: procalcitonin; GGO: ground glass opacities; NRM: non-rebreather mask; NC: nasal cannula; SOFA: Sequential Orfan Failure Assessment; BVAS: Birmingham Vasculitis Severity; MP: methylprednisolone; RTX: rituximab; AAV: ANCA-associated vasculitis; RBC/hpf: red blood cells per high-power field; TD: total dose; Urinalysis (E, LE, Prot) was performed by dipstick method and the grading system is as follows: "negative", trace ("+/-"), positive/detectable ("+"), moderate ("++"), high grade ("+++").
